# Implications of White Light-Emitting Diode-Based Photoirradiation on Green Synthesis of Silver Nanoparticles by Methanol- and Aqueous-Based Extracts of *Bergenia ciliata* Leaves

**DOI:** 10.3390/nano14161327

**Published:** 2024-08-07

**Authors:** Sourav Gurung, Monalisha Sarmin, Muddasarul Hoda

**Affiliations:** Nanobiotechnology and Applied Phytochemistry Lab, Department of Biological Sciences, Aliah University, Kolkata 700160, India; gsouravee@gmail.com (S.G.); m.s1rmin@gmail.com (M.S.)

**Keywords:** photoirradiation, *Bergenia ciliata*, green synthesis, silver nanoparticles, nanoparticle stability

## Abstract

*Bergenia ciliata* (BC) is a perennial herb that is frequently used as a traditional medicine. Its leaves and rhizomes are reported to have significant antioxidant, metal-reducing, and chelating properties. Although the rhizomes have the potential to synthesize silver nanoparticles (AgNPs), the leaves are yet to be studied for the green synthesis of metal nanoparticles. Likewise, photoirradiation also plays a significant role in the green synthesis of metal nanoparticles. In the current study, we intended to demonstrate the implications of photoirradiation by white light-emitting diode (LED) on the aqueous and methanol extracts (AE and ME, respectively) of BC leaf-mediated green synthesis of AgNPs. In this regard, the AgNP synthesis of the two extracts was performed in the dark and under 250-lumen (lm) and 825 lm LED bulbs. The physicochemical characterization of the synthesized nanoparticles was also performed, wherein percent nanoparticles yield, morphology of the nanoparticles, shape, size, percent elemental composition, crystallinity, and nanoparticle stability were studied. The nanoparticle-synthesizing potential of the two extracts contradicted significantly in the presence and absence of light, while the AE produced a significantly high number of nanoparticles in the dark (17.26%), and increasing light intensities only attenuated the nanoparticle synthesis, whereas ME synthesized comparatively negligible silver nanoparticles in the dark (1.23%). However, increasing light intensities significantly enhanced the number of nanoparticles synthesized in 825 lms (7.41%). The GCMS analysis further gave a comparative insight into the phytochemical composition of both extracts, wherein catechol and pyrogallol were identified as major reducing agents for nanoparticle synthesis. The influence of light intensities on the physiochemical characterization of AgNPs was predominant. While the size of both the AE- and ME-mediated AgNPs increased considerably (20–50 nm diameter) with increasing light intensities, the percent of silver atoms decreased significantly with increasing light intensities in both the AE- and ME-mediated AgNPs with ranges of 13–18% and 14–24%, respectively. The nanoparticle stability studies suggested that both the AE- and ME-mediated AgNPs were stable for up to 15 days when stored at 4 °C. The stability of both nanoparticles was attributed to the presence of a wide range of phytochemicals. In conclusion, the AE of BC leaves was reported to have significantly higher AgNP-synthesizing potential as compared to the ME. However, AE-mediated AgNP synthesis is attenuated by photoirradiation, whereas ME-mediated AgNP synthesis is enhanced by photoirradiation. The photoirradiation by white LED light increases the size of the AgNPs, while the percent silver composition declines, irrespective of the extract type. Both extracts, however, have nanoparticle stabilizing potential, thereby producing stable nanoparticles.

## 1. Introduction

Silver nanoparticles (AgNPs) have garnered significant attention in recent years, owing to their exceptional physicochemical properties, including augmented optical and electrical conductivity, robust antimicrobial and antiviral activities, and enhanced Raman scattering capabilities [1,2,3]. Beyond their applications in the biomedical field, AgNPs have demonstrated significant potential in the textile industry, where they are utilized to impart antimicrobial properties to fabrics, and in the wastewater treatment sector, where they exhibit enhanced catalytic activity for pollutant degradation, highlighting their versatility and value in diverse industrial domains [4].

The synthesis of AgNPs typically involves the reduction of silver ions (Ag^+^) to silver atoms (Ag^0^), which can be achieved through various methods, including chemical, physical, and biological processes [5]. Notably, green synthesis of AgNPs has emerged as a safer and cost-effective approach, leveraging plant products or microorganisms as precursors [6]. A plethora of studies have exploited plant extract-based synthesis of AgNPs due to their wide range of phytochemicals, comprising alkaloids, flavonoids, phenolic acids, carbohydrates, lipids, pigments, alcohols, vitamins, and amino acids [7]. These phytochemicals play a pivotal role in reducing, capping, and stabilizing the AgNPs, thereby facilitating their synthesis [8,9]. However, the optimization of the phytochemical composition of the plant extract is a major challenge that, in turn, depends on the extraction parameters. As a result, it is difficult to identify the most probable compounds that contribute to nanoparticle synthesis. The prevailing theory suggests that electrostatic interactions between silver ions (Ag^+^) and bioactive compounds in plant extracts trigger a redox process, although the intricate mechanistic pathways and specific molecular entities involved in plant-mediated nanoparticle synthesis remain partially unresolved, warranting further investigation [10].

*Bergenia ciliata* (BC), a perennial herb native to the lower Himalayan regions, has been traditionally utilized by local communities for various traditional medicinal properties. Belonging to the Saxifragaceae family, BC leaves and rhizomes have traditionally been used to treat wounds, cuts, colds, coughs, and kidney stones for centuries. Phytochemical analysis of various extracts of the plant has revealed the presence of bioactive compounds, such as benzoic acid, gallic acid, quercetin, pyrogallol, and catechol, all of which contribute to the medicinal properties of BC [11]. The diverse composition of these phenolic compounds contributes to the immense reducing and antioxidant potential of BC that may be exploited for nanoparticle synthesis. The rhizome extract of BC has been reported to have the potential to synthesize medicinally significant silver nanoparticles [12]. However, BC leaves have not yet been explored for green synthesis of silver nanoparticles. Our previous unpublished study has also confirmed its antioxidant properties, which are attributed to its high phenolic and flavonoid content. Given its exceptional antioxidant potential, we intend to explore BC leaves’ potential in the green synthesis of AgNPs. A silver nanocomposite of BC leaves may have significant therapeutic and biomedical applications.

Photoirradiation plays a significant role in green synthesis of metal nanoparticles. Photoirradiation of experimental setup by a wide range of electromagnetic radiation, including gamma, ultraviolet, and microwave, has been reported to enhance the rate of nanoparticle synthesis [13,14,15]. Light-emitting diode (LED) as a source of photoirradiation in green synthesis of AgNPs has previously been reported [16]. Likewise, light intensity also has a significant impact on metal nanoparticle synthesis [17]. In this context, the current study intends to demonstrate the AgNP-synthesizing potential of the aqueous and methanol extracts (AE and ME, respectively) of BC leaves, in addition to studying the implications of white LED-based photoirradiation on green synthesis and physicochemical characterization of AgNPs.

## 2. Materials and Methods

### 2.1. Materials

Leaves of BC were collected from the foothills of Mungpoo, in the district of Darjeeling, India (26°59′38.8″ N 88°20′43.3″ E), in early February during the flowering season and were identified by the Central National Herbarium, Botanical Survey of India, Howrah, India. Silver nitrate (AgNO_3_, CAS No. 030087) of analytical grade was purchased from Central Drug House (P) Ltd., Mumbai, India. All experiments were conducted utilizing double-distilled water as the solvent.

### 2.2. Extraction of Phytochemicals from Bergenia ciliata by Maceration

The leaves were thoroughly rinsed with distilled water and non-ionic detergent; excessive water was drained off, and the leaves were left to dry overnight at 40 °C in a hot air oven. The dried leaves were wholly crushed and ground into a fine powder using a mixer grinder. The fine powder was sealed with parafilm and stored at 4 °C in air-tight containers until further use. Extraction of phytochemicals from the leaf powder was performed by a maceration technique wherein two separate polar solvents, i.e., methanol and water, were used for the extraction process, thereby resulting in the production of two separate extracts, namely ME and AE. In brief, 2 g of leaf powder was added to 50 mL of solvents and kept on a magnetic stirrer at 700 rpm for 24 h in amber bottles at room temperature. Subsequently, methanol-based macerated extracts were filtered by Whatman filter paper no. 1, and the filtrate was concentrated by rotary evaporator (make: Eyela^®^, Tokyo, Japan; model: N-1300 V-W) under reduced pressure in a 40 °C water bath. The concentrated extract was further dried in a hot-air oven at 40 °C, until dry. Likewise, the aqueous-based maceration mixture was centrifuged at 6000 rpm for 10 min at 25 °C. The supernatant, which consisted of dissolved phytochemicals and other metabolites, was isolated and subsequently dried in a hot-air oven at 40 °C until dry.

### 2.3. Gas Chromatography-Mass Spectroscopy (GCMS) Analysis of the Extract

The sample was dissolved in methanol and injected into a GC–MS QP2010 model (Shimadzu^®^, Kyoto, Japan) column, GC, SH-I-5Sil MS Capillary, 30 m × 0.25 mm × 0.25 um, injection mode: Spitless. The operating conditions of the GC–MS set for the analysis were as follows: oven temperature 45 °C for 2 min, then 140 °C at 5 °C/min, and finally, increased to 280 °C and held isothermally for 10 min. The sample injection was 2 μL, and the carrier gas was helium at 1 mL/min. The ionization of the sample components was conducted at 70 eV. The running time of the GC was from 9.10 to 52.0 min. The GC–MS data were analyzed using the NIST14.L library (2020) to identify the phytochemical components by comparing the retention time and mass spectra of the predicted compounds with those of the NIST database.

### 2.4. Optimization of Green Synthesis of Silver Nanoparticle Synthesis

AgNPs was synthesized by exploiting the reducing potential of the extracts. To determine the optimum extract concentration, 10 mL of 1 mM AgNO_3_ solution was mixed with 1 mL each of the extract solution at varying concentrations. The mixture was then incubated at 25 °C under ambient light conditions. A double-beam UV–visible spectrophotometer (make: Shimadzu^®^, Kyoto, Japan; model: UV-1900i) was used to measure the absorbance spectrum at specific intervals for up to five hours from the initial observation of nanoparticle formation.

### 2.5. Effect of White Light-Emitting Diode on the Green Synthesis of Silver Nanoparticles

Three separate sets with 1 mL of extract (1 mg mL^−1^) each were dissolved in 10 mL each of 1 mM AgNO_3_. The solutions were incubated with an exposure time of one hour each under three different light intensities, i.e., dark, 250 lumens (lms) and 825 lms, respectively. A constant temperature of 25 °C was maintained in each set under a BOD incubator. At specific time intervals, UV–visible spectral analysis was performed to monitor the rate of change of λ_max_ with a UV–visible spectrophotometer (make: Shimadzu^®^, Japan; model: UV-1900i) using a quartz cuvette.

### 2.6. Characterization of the Silver Nanoparticles

#### 2.6.1. Percent Nanoparticle Yield and Particle Size Analysis of the Silver Nanoparticles Synthesized by the Extracts

The nanocolloidal solutions were analyzed by a dynamic light-scattering technique (make: Malvern^®^, Malvern, UK; model: ZetaSizer^®^) to confirm the particles size range of the nanoparticles. The nanocolloidal solution was centrifuged at 21,000 rcf (Eppendorf^®^, Hamburg, Germany; model: 5424R), and the pelleted nanoparticles were dried. Subsequently, the net weight of the nanoparticles was measured by analytical balance (make: Sartorius^®^, Göttingen, Germany; model: QUINTIX224-10IN) and the percent nanoparticle yield was calculated by the formula:

(1) $$Percent\;nanoparticles\;yield\left(\%\right)=\frac{W_{1}}{W_{2}}\times 100$$

*W*_1_ = weight of silver nanoparticles; *W*_2_ = initial weight of the extract and silver nitrate.

#### 2.6.2. Morphological Study of the Nanoparticles by Scanning Electron Microscopy

The morphology of the nanoparticles synthesized by the AE and ME, respectively, was studied by scanning electron microscopy (SEM) (make: Carl Zeiss^®^, Oberkochen, Germany). A 10 μL of 100× dilution of the nanocolloidal solutions was added onto 1 cm^2^ glass slides, and dried in a desiccator. Prior to sample loading, the nanoparticle-mounted glass slides were coated by gold coating. The SEM images were captured at 50–75k× magnification and 15 kV voltage.

#### 2.6.3. Elemental Analysis of the Nanoparticles by Energy-Dispersive X-ray (EDX) Spectroscopy

Elemental analysis of the nanoparticles was conducted using energy-dispersive X-ray spectroscopy (EDX), a module integrated into the SEM (make: Carl Zeiss^®^, Oberkochen, Germany). This technique exploits the unique outer electron configuration of each element, which can be excited by X-ray irradiation, causing electrons to transition to higher energy orbitals. As these electrons return to their ground state, they release excess energy, which is detected and analyzed to identify and quantify the elemental composition of the nanoparticles. This analytical approach enables the determination of the elemental makeup of the nanoparticles with high precision.

#### 2.6.4. Functional Group Analysis of the Nanoparticles by Fourier Transform Infrared (FTIR) Analysis

Fourier transform infrared (FTIR) spectroscopy was employed to investigate the surface functional groups of the synthesized nanoparticles and a physical mixture of extract and AgNO_3_ powder. The FTIR spectra were recorded over a frequency range of 4000–400 cm⁻^1^ using a FTIR spectrometer (make: PerkinElmer^®^, Singapore; make: Spectrum 100). The extracts and the AgNPs were individually mixed in KBr powder, and pellets were made by hydraulic press. This analytical technique enabled the identification of various functional groups present on the nanoparticle surface, providing valuable insights into the chemical properties and potential interactions of the nanoparticles.

#### 2.6.5. X-ray Diffraction Analysis

The crystalline structure of the synthesized AgNPs was determined and confirmed by X-ray diffraction (XRD) analysis. The centrifuged and air-dried AgNPs were placed on a glass microscope slide and analyzed using a X-ray diffractometer (make: BRUKER^®^, Ettlingen, Germany; model: D8). The average crystallite size of the synthesized AgNPs was calculated using Scherrer’s formula:

(2) $$D=\frac{\left(0.89\lambda \right)}{\left(\beta cos\theta \right)}$$

where *D* is the average crystallite size; *λ* is the incident wavelength (0.154056 nm); *β* is the full width at half maximum; and *θ* is the Bragg’s diffraction angle [18].

#### 2.6.6. Stability Studies of Nanoparticles

The nanoparticles synthesized by one hour of exposure time were collected and stored at 4 °C for further analysis. Spectra analysis was performed at regular time intervals over a 15-day period, with absorbance spectra recorded in the wavelength range of 350–700 nm. This allowed us to monitor the stability of the nanoparticles over time. Additionally, we compared the stability of the synthesized nanoparticles using both extracts under different light conditions, where they had previously shown optimal nanoparticle synthesis. This comparison enabled us to assess the effects of extract type on nanoparticle stability.

### 2.7. Statistical Analysis

All the experiments were performed in triplicates and were presented as mean ± SD of the observations. All the data were analyzed using the OriginPro^®^ software (8.5.0 SR1 version). Statistical analysis was conducted by analysis of variance (ANOVA) followed by Tukey’s *t*-test, where *p* ≤ 0.05 was statistically significant.

## 3. Results

AE and ME obtained from BC leaves by the maceration technique yielded a significantly diverse range of phytochemicals. The total phenolic and flavonoid contents were previously estimated for both AE and ME (unpublished data) (Table 1). The extracts also demonstrated significantly high cation-reducing activity, with the ME having higher cation-reducing power as compared to that of AE (unpublished data). The metal-ion-reducing activities of the extracts were explored to study their potential in metal nanoparticle synthesis.

### 3.1. Gas Chromatography–Mass Spectroscopy (GCMS) Analysis of the Extract

The GCMS data from both the extracts revealed distinct spectral patterns, with each extract containing a wide range of phytochemicals, some of which were available in both the extracts, albeit in varying concentrations. A broad range of secondary metabolites, including phenolic acids, carbohydrates, lipids, amino acids, and vitamins, was identified in both extracts. Notably, the ME contained a more diverse range of phytochemicals compared to the AE. After manual curation, the GCMS data identified 15 bioactive phytochemicals in the AE (Table 2) and 18 in the ME (Table 3). Interestingly, nine of these bioactive phytochemicals were present in both the extracts, albeit in different concentrations. The most abundant phytochemicals common to both extracts were pyrogallol, catechol, hydroquinone, benzoic acid, and 5-hydroxymethylfurfural. In addition to the bioactive phytochemicals, Table 2 and Table 3 also include a comprehensive list of compounds that play a crucial role in nanoparticle synthesis, specifically those that function as reducing agents, stabilizing agents, and capping agents. These compounds are essential for the formation, growth, and stabilization of nanoparticles, and their presence in both the AE and ME is noteworthy.

### 3.2. Concentration-Dependent Optimization of Silver Nanoparticle Synthesis

**Figure 1:**
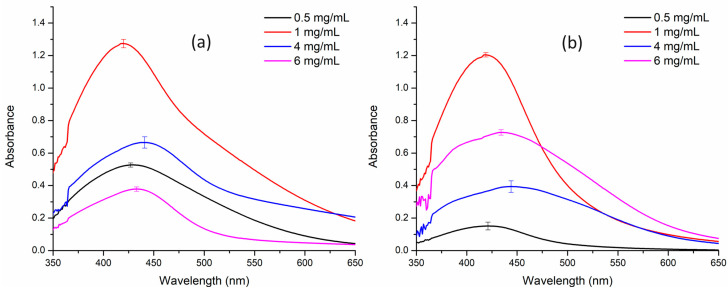
Concentration-dependent optimization of silver nanoparticle synthesis. (**a**) Aqueous extract; (**b**) methanol extract.

Optimization studies revealed that a concentration of 1 mg/ml of extract was ideal for the synthesis of AgNPs in combination with 1 mM AgNO_3_. Consequently, all subsequent characterization experiments were conducted using the optimized concentration of AgNO_3_ and the extract, ensuring the most favorable conditions for nanoparticle synthesis and analysis (Figure 1).

### 3.3. Effect of White Light-Emitting Diode on the Green Synthesis of Silver Nanoparticles

**Figure 2:**
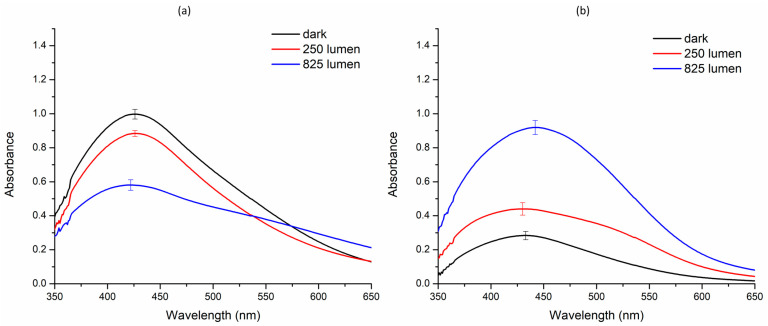
Effect of various intensities of white light-emitting diode on the green synthesis of silver nanoparticles. (**a**) Aqueous extract; (**b**) methanol extract.

The green synthesis of silver nanoparticle was investigated under three distinct light conditions: dark, 250 lms, and 825 lms. The results varied significantly across these setups. AE-based nanoparticles exhibited optimum synthesis in the dark, with a decrease in the nanoparticle synthesis observed at 250 lms and 825 lms, respectively. In contrast, ME-based nanoparticles displayed minimal synthesis in the dark, but the synthesis increased at 250 lms and reached a maximum at 825 lms (Figure 2).

### 3.4. Characterization of the Silver Nanoparticles

#### 3.4.1. Percent Nanoparticle Yield and Particle Size Analysis of the Silver Nanoparticles Synthesized by the Leaf Extracts

**Figure 3:**
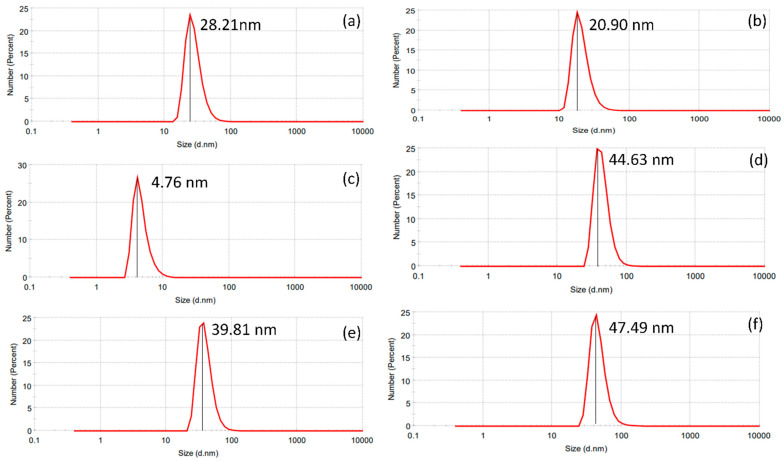
Particle size analysis of aqueous and methanol extracts by dynamic light scattering technique. (**a**) Aqueous extract under darkness; (**b**) methanol extract under darkness; (**c**) aqueous extract under 250 lms; (**d**) methanol extract under 250 lms; (**e**) aqueous extract under 825 lms; (**f**) methanol extract under 825 lms.

The nanoparticle-yielding potential for both AE and ME was estimated under various light conditions. In the dark, the nanoparticle yield by AE and ME were estimated to be 17.26% ± 0.06 and 1.23% ± 0.06, respectively, whereas, under 250 lms, the nanoparticle yield was 12.04% ± 0.57 for AE and 4.52% ± 0.141 for ME, respectively, while, under 825 lm LED illumination, the nanoparticle yield by AE and ME were estimated to be 8.63% ± 0.57 and 7.41% ± 0.14, respectively. The results suggest a significant difference in the nanoparticle-yielding potential of AE and ME under various light intensities (Table 4). Dynamic light scattering analysis revealed a range of nanoparticle sizes under varying light intensities (Table 4). Notably, both AE- and ME-based AgNPs exhibited a similar pattern of considerable increase in the mean diameter of the nanoparticles with increasing light intensities (Figure 3).

#### 3.4.2. Morphological Study of the Nanoparticles by Scanning Electron Microscopy

**Figure 4:**
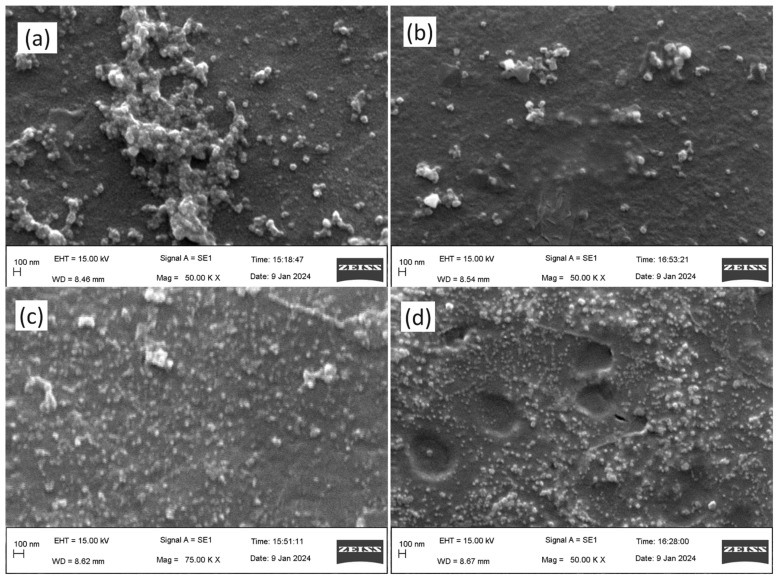
Morphological study of the silver nanoparticles by scanning electron microscopy. (**a**) Aqueous extract under darkness; (**b**) methanol extract under darkness; (**c**) aqueous extract under 825 lms; (**d**) methanol extract under 825 lms.

SEM analysis revealed the morphology of the AgNPs, which exhibited similar features across all samples. Notably, both AE- and ME-based AgNPs displayed a spherical shape, confirming their uniform morphology. Furthermore, the microscopic images correlated well with the particle size measurements obtained from DLS analysis, with the observed sizes aligning closely with the diameters measured by DLS. This consistency in size and shape across different analytical techniques supports the reliability of the characterization results (Figure 4).

#### 3.4.3. Elemental Analysis of Nanoparticles by Energy-Dispersive X-ray (EDX) Spectroscopy

**Figure 5:**
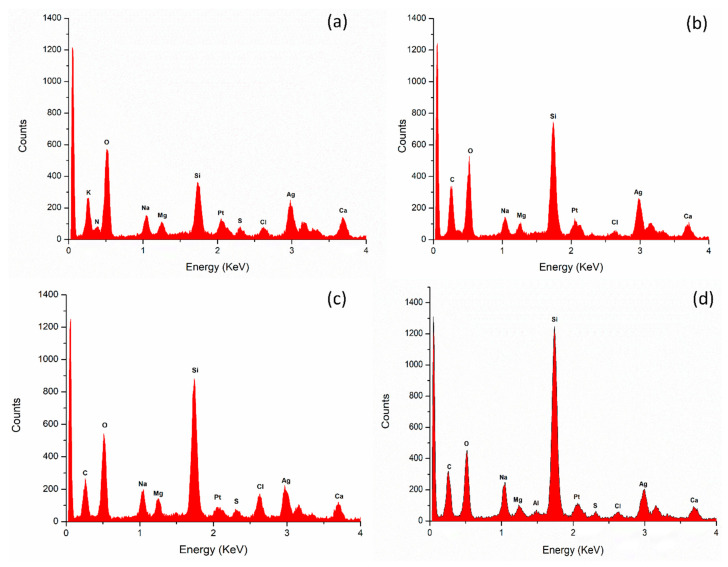
Elemental analysis of nanoparticles by energy-dispersive X-ray (EDX) spectroscopy. (**a**) Aqueous extract under darkness; (**b**) methanol extract under darkness; (**c**) aqueous extract under 825 lms; (**d**) methanol extract under 825 lms.

EDX spectroscopy analysis revealed the percentage of a diverse range of elements in the nanoparticle samples. Notably, silver (Ag) was consistently present in the highest abundance across all cases (Table 5). Additionally, other elements, such as platinum (Pt), magnesium (Mg), calcium (Ca), and chlorine (Cl), were also detected, albeit in smaller quantities. The presence of these elements suggests a complex composition of the nanoparticles, with Ag being the dominant component (Figure 5).

#### 3.4.4. Functional Group Identification of the Silver Nanoparticles by Fourier Transform Infrared Analysis

**Figure 6:**
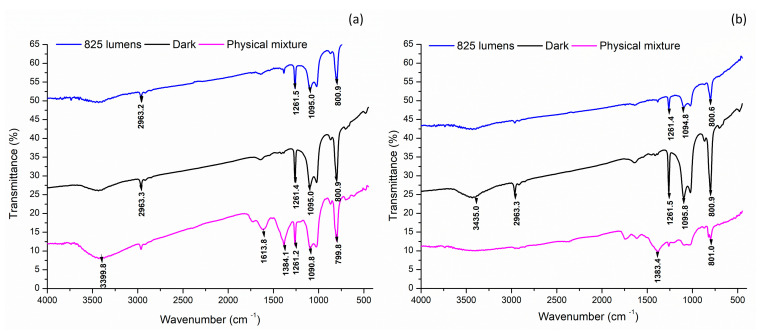
Functional group identification of the silver nanoparticles by Fourier transform infrared (FTIR) analysis. (**a**) Aqueous extract; (**b**) methanol extract.

FTIR spectroscopy analysis of the AgNPs synthesized under various light conditions revealed the presence of distinct bonding arrangements (Figure 6).

A comparative analysis of the FTIR spectra showed that AE-based nanoparticles exhibited consistent peaks at wavenumbers 1261, 1095, and 800.9 cm⁻^1^ across all light conditions, including the physical mixture. In contrast, ME-based nanoparticles displayed a common peak only at 800.7 cm⁻^1^, indicating a difference in bonding patterns between the two types of nanoparticles (Table 6) [38]. These findings suggest that the bonding arrangements in the nanoparticles are influenced by the extract used and the light conditions employed during synthesis.

#### 3.4.5. X-ray Diffraction (XRD) Analysis

**Figure 7:**
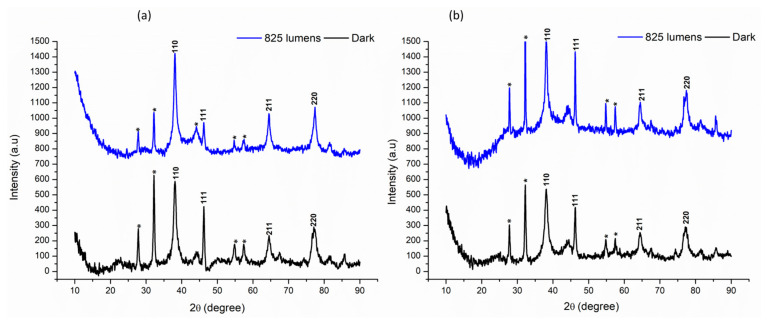
Comparative X-ray Diffraction (XRD) analysis under darkness and 825 lumens of light intensity. (**a**) Aqueous extract-based silver nanoparticles; (**b**) methanol extract-based silver nanoparticles. * signifies the peaks that are not reported in XRD JCPDS value of silver.

XRD analysis of the nanoparticles revealed similar patterns in both AE- and ME-based nanoparticles across all light conditions. The diffraction peaks were compared to the standard powder diffraction card of JCPDS (silver file No. 04-0783) and showed a match at 2θ values of 38.17, 46.19, 64.60, and 77.18, corresponding to hkl values of (110), (111), (211), and (220), respectively. These peaks confirm the presence of silver in the nanoparticles. Additionally, a few unidentified peaks were observed at other 2θ values, as shown in Figure 7. The average crystallite size of the AgNPs was calculated to be approximately 8–10 nm, indicating a nanocrystalline structure (Figure 7).

#### 3.4.6. Stability Studies of the Silver Nanoparticles

**Figure 8:**
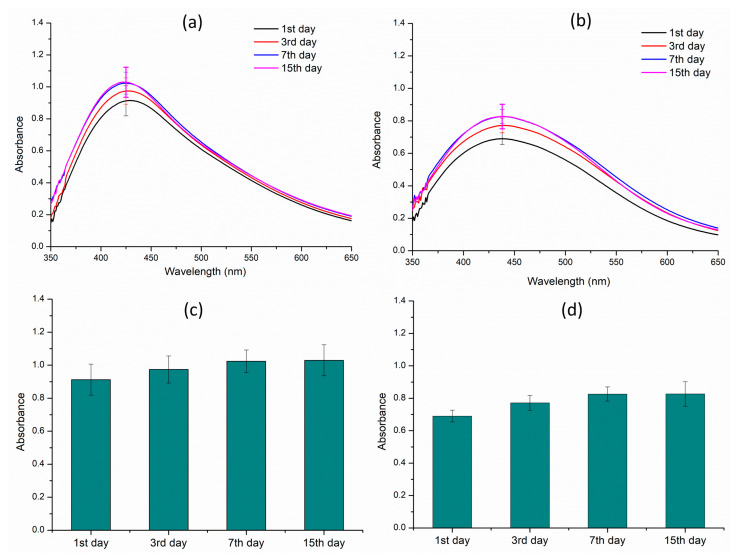
Stability studies of the silver nanoparticles over a period of 15 days. (**a**) Aqueous extract; (**b**) methanol extract; (**c**) λmax of the aqueous extract; and (**d**) λmax of the methanol extract.

A comprehensive stability study was conducted to assess the shelf life of both AE- and ME-based nanoparticles over a period of 15 days. During the entire incubation time, absorption spectra analysis was performed at various time intervals to detect any changes in the absorption intensity that indicate degradation of the nanoparticles. A comparison between the spectra of various time periods for both types of nanoparticles indicated excellent stability, with no significant changes in their spectral profiles observed throughout the 15-day period (Figure 8). This indicates that the nanoparticles remained structurally intact and retained their physicochemical properties, demonstrating a high level of stability and robustness. The stability of the nanoparticles is a crucial factor in their potential applications, and this study suggests that both AE- and ME-based nanoparticles potentially have the stability that may be required for various applications.

## 4. Discussion

BC is a plant of unparalleled versatility, renowned for its multifaceted properties that make it a valuable resource of therapeutic importance. Its various plant parts, including leaves, rhizomes, and roots, possess significant medicinal importance, and it also serves as an ornamental plant, adding beauty to its functional value. Despite its immense potential, surprisingly few studies have been conducted on BC, with existing research primarily focusing on its antioxidant potential. However, all these studies have consistently reported BC to be a potent antioxidant and reducing agent, capable of neutralizing free radicals and mitigating oxidative stress [39]. This high antioxidant and metal-reducing potential of BC leaves makes it an ideal candidate for mediating green synthesis of AgNPs, which have numerous applications in biomedical and nanotechnology fields.

Previously, several studies have reported green synthesis of nanoparticles using crude extracts of the rhizomes and roots, but these investigations lacked critical parameters, such as light conditions, temperature, and pH, which are essential for optimization of NP synthesis [12]. In this pioneering study, we have successfully demonstrated synthesis of AgNPs from BC leaf extract under various light conditions, a novel approach that expands our understanding of this plant’s potential applications and opens new avenues for nanoparticle research. Initially, concentration-dependent optimization of both AE and ME against 1 mM AgNO_3_ was performed. It was observed that the optimum concentration of both extracts was 1 mg mL^−1^ (Figure 1). Following the concentration optimization, the influence of light on nanoparticle synthesis was demonstrated, the outcome of which was contrasting for AE and ME (Figure 2).

During the ME-mediated nanoparticle synthesis, it was observed that there was negligible nanoparticle synthesis in the dark, whereas the AE demonstrated a significantly high percentage of nanoparticle synthesis, contrary to that of the ME. This preliminary observation led to the second objective, i.e., studying the impact of photoirradiation on the rate of silver nanoparticle synthesis under various light intensities. Three independent experimental setups with different light intensities were compared for percent nanoparticle yield. Initially, the nanoparticle synthesis mediated by individual extracts was performed in the dark, wherein the AE demonstrated a significantly high nanoparticle yield, while the ME demonstrated a negligible nanoparticle yield. We further performed similar experiments under two more light intensities, i.e., 250 lms and 825 lms, for both extracts. Interestingly, the ME demonstrated an increase in the rate of nanoparticle synthesis with increasing light intensities, whereas the AE demonstrated the opposite trend, i.e., the nanoparticle synthesis was attenuated with increasing light intensities. The results of the percent nanoparticle yield corroborated with the spectra analysis (Figure 2). This disparity in the nanoparticle yield between the two extracts highlights the significance of light intensity as an important parameter in nanoparticle synthesis. In the context of the influence of light on nanoparticle synthesis, few reports suggest sunlight induces green synthesis of AgNPs [40,41]. However, none of the reports have attempted to identify the reason behind this observation. Likewise, the problem with sunlight is that it is difficult to maintain the uniformity of photoirradiation, thus contributing to the issue of reproducibility of the experimental data, whereas lab-controlled light exposure such as ours has a significantly higher probability of reproducibility. The AE-mediated AgNP synthesis, on the other hand, gets attenuated on exposure to light. The probable reason for this attenuation is not known, as there is no reported study on attenuation of green synthesis of nanoparticles. This lacunae in research reports has become the objective of our follow-up research project, on which we are currently working.

The GC–MS analysis of both the extracts identified various types of phenolic compounds and fatty acids. Pyrogallol and catechol were observed to be predominantly present in both extracts. However, ME had significantly higher pyrogallol percentage as compared to the AE, whereas the catechol percentage was higher in the AE. Since the silver nanoparticle-synthesizing potential of the AE was observed to be significantly higher than that of the ME, both in dark and light conditions, we may infer that catechol may be a stronger reducing agent than pyrogallol, and hence may be the major contributor in the reducing process during the nanoparticle synthesis.

The third objective of the study was to investigate the impact of photoirradiation with different intensities on the physicochemical characteristics of the synthesized nanoparticles. To address this objective, we performed DLS analysis to determine the mean particle size of all nanoparticles produced under various light intensities. Our findings revealed a fascinating trend wherein the particle size of nanoparticles synthesized by ME increased proportionally with increasing light intensities ranging from 48.43 nm in dark conditions to 75.19 nm under 825 lms. Similarly, the particle size of AE-mediated AgNPs increased from 55.53 nm in dark conditions to 66.93 nm in 825 lms, suggesting that the light intensity may have the property of increasing the particle size, regardless of the rate of nanoparticle synthesis. This observation has significant implications on nanoparticle synthesis with specific characteristics, as it highlights the potential for light intensity to be used as a parameter to control the particle size. The influence of photoirradiation on the size of the nanoparticles was also observed during the green synthesis of magnesium oxide nanoparticles by Siaw et al. 2020, thus corroborating our finding [42]. However, the morphology of the nanoparticles was thoroughly examined using SEM, which confirmed that all nanoparticles exhibited a spherical shape, and photoirradiation does not have any major influence on the morphology of the nanoparticles.

The elemental composition of the nanoparticles is an important parameter to investigate. Photoirradiation significantly influences the percentage of the silver atoms in the nanoparticles. This was demonstrated in our EDX data, wherein we compared the elemental composition of both AE- and ME-mediated AgNPs that were synthesized under dark and light conditions. Notably, in ME-mediated AgNPs, the percentage of silver atoms was estimated to be 21.166 ± 5.25% in darkness and 14.166 ± 2.34% in 825 lms of light intensity, indicating a significant decline in the silver atom deposition in the nanoparticles. Likewise, the percentage of silver atoms in AE-mediated AgNPs was estimated to be 18.16 ± 1.011% in darkness and 13.8 ± 0.78% in 825 lms of light intensity. However, in our previous reports, it was observed that the percentage of silver atoms considerably increased with increasing light intensities in both AE-mediated and ME-mediated AgNPs synthesized by fenugreek seeds [17,43]. Thus, these contradicting outcomes of BC leaves and fenugreek seeds suggest that the influence of photoirradiation is plant source-specific and requires further experiments to get a comprehensive insight into the influence of photoirradiation on the elemental composition of AgNPs. Furthermore, our analysis revealed the presence of other elements, including platinum (Pt), magnesium (Mg), calcium (Ca), chlorine (Cl), carbon (C), nitrogen (N), and oxygen (O), which may be attributed to the presence of various phytochemicals and biomolecules in the leaf extract.

The presence of various functional groups plays a significant role in the formation of different types of bonded and non-bonded interactions in the nanoparticles, thereby attributing stability and characteristic properties to the nanoparticles [44]. The presence of C-H bonds in all AgNPs indicates the presence of alkanes, which can be attributed to the alkyl group of various fatty acids and their esters, such as n-hexadecanoic acid, hexadecanoic acid, octadecanoic acid, 2-hydroxy-1-(hydroxymethyl) ethyl ester, and 2,3-dihydroxypropyl ester. Similarly, the C-O stretching vibrations reveal the presence of different carboxylic acids, including benzoic acid, and cis-cinnamic acid. Furthermore, the O-H and N-H stretching vibrations indicate the presence of various amino acids. Overall, the FTIR study reveals that despite differences in light intensities, all AgNPs exhibited the presence of similar types of bonds, suggesting a common underlying chemical structure. This finding provides valuable insight into the composition and properties of NPs, which is essential for understanding their behavior and potential applications.

The XRD study revealed a consistent result across all AgNPs, explicitly suggesting that all the synthesized AgNPs have a crystalline character. The presence of four distinct peaks at 2θ values of 38.17, 46.19, 64.60, and 77.18 is attributed to the presence of silver metal, as our results align with the Joint Committee on Powder Diffraction Standards (JCPDS) value for silver, thus indicating a high degree of crystallinity in the AgNPs [45]. However, in addition to these characteristic silver peaks, few other peaks were also observed at 2θ values of 27, 32, 54, 57, 64, and 85. These additional peaks were identified to correspond to Ag^+^ ions, which may not have been reduced during the process of nanoparticle synthesis, and thus remained in the AgNPs in minute quantities [46]. The presence of the residual silver ions peaks suggests that the reduction reaction may have reached equilibrium towards completion of the reaction, leaving behind traces of unreacted silver ions in the final product [47].

A nanoparticle stability study was also conducted for both AE- and ME-mediated AgNPs over a 15-day period, which revealed considerable stability of the nanoparticles in both the cases. This notable stability can be attributed to the presence of various bioactive compounds, which not only function as reducing agents but also as stabilizing agents, thereby preventing the aggregation and sedimentation of the nanoparticles. The presence of these bioactive compounds, which include phenolic acids, flavonoids, and amino acids, among others, plays a crucial role in maintaining the stability and integrity of the AgNPs. Furthermore, the details of the specific phytochemicals serving as stabilizing agents are also reported in our study, as curated in Table 2 and Table 3, thus providing a comprehensive understanding of the composition and properties of these stable AgNPs. The stability of these nanoparticles is a critical factor in their potential applications, and this study demonstrates the stability of these AgNPs for use in various fields, including biomedicine and catalysis.

## 5. Conclusions

The leaf extract of BC possesses excellent metal-reducing and nanoparticle-stabilizing properties, which are attributed to the high concentration of polyphenols and flavonoids, which enables efficient reduction of silver ions. Upon thorough investigations, it is evident that light played a significant role in controlling the rate of nanoparticle synthesis, with the ME exhibiting a direct correlation with increasing light intensities, whereas the AE displayed an inverse relationship. Notably, despite the disparity in nanoparticle synthesis rates between the two extracts, light exhibited a distinct impact in controlling the particle size and percentage of silver atoms in the silver nanoparticles of both extracts. Moreover, the crystalline nature and significant stability of both AE- and ME-based AgNPs were demonstrated to be comparable. This study highlights the significance of photoirradiation in influencing the rate and characterization of nanoparticles under different light intensities. However, it also suggests that photoirradiation alone cannot account for the differences in nanoparticle synthesis rates between solvent-based extracts, indicating the need for further research to elucidate the underlying reasons for this variation. This finding opens avenues for future investigations into the complex interactions between solvent-based extracts, photoirradiation, and nanoparticle synthesis.

## Figures and Tables

**Table 1:** Estimation of total phenolic content and total flavonoid content of the extracts.

Extract Type	Total Phenolic Content (mg * QE/g of Extract)	Total Flavonoid Content (mg * QE/g of Extract)
Methanol	176.553 ± 0.003	116.512 ± 0.002
Aqueous	111.067 ± 0.035	82.218 ± 0.001

* QE = quercetin equivalent.

**Table 2:** Gas chromatography–mass spectroscopy of the aqueous extract of *Bergenia ciliata* leaves.

S. No.	Chemical Name	Type of Phytochemical	PubChem ID	Area%	Reducing Potential	Stabilizing Activity
01.	Pyrogallol	phenol	1057	20.75	[19]	[20]
02.	5-Hydroxymethylfurfural	furan	237332	12.00	--	--
03.	Catechol	phenol	289	8.61	[21]	[22]
04.	13-Docosenamide, (Z)-	fatty amide	5365371	2.49	--	--
05.	Hydroquinone	phenol	785	2.19	[23]	[24]
06.	2-Palmitoylglycerol	fatty acid ester	123409	0.79	--	--
07.	palmitic acid	fatty acid	985	0.35	--	[25]
08.	Phenol	phenol	996	0.29	[26]	--
09.	Benzoic acid	carboxylic acid	243	0.18	[27]	[28]
10.	Glyceryl monostearate	fatty acid ester	24699	0.18	--	[29]
11.	4-Vinylphenol	phenol	62453	0.15	[26]	[30]
12.	myristic acid	fatty acid	11005	0.14	--	[31]
13.	Squalene	terpenoid	638072	0.13	--	--
14.	Geranic acid	fatty acid	5275520	0.08	--	--
15.	dihydroferulic acid	carboxylic acid	14340	0.06	--	--

**Table 3:** Gas chromatography–mass spectroscopy of the methanol extract of *Bergenia ciliata* leaves.

Sl. No.	Chemical Name	Type of Phytochemical	Pubchem ID	Area %	Reducing Potential	Stabilizing Activity
01.	Pyrogallol	phenol	1057	38.85	[19]	[20]
02.	5-Hydroxymethylfurfural	furan	237332	9.17	--	--
03.	Hydroquinone	phenol	785	5.78	[24]	[24]
04.	palmitic acid	fatty acid	985	4.38	--	[25]
05.	Vitamin E	vitamin	483926503	3.14	[32]	[32]
06.	myristic acid	fatty acid	11005	0.96	--	[31]
07.	methyl palmitate	fatty acid ester	8181	0.90	--	[33]
08.	1-Dodecanol	fatty alcohol	8193	0.69	[34]	[34]
09.	Catechol	phenol	289	0.67	[21]	[22]
10.	linoleic acid	fatty acid	5280450	0.47	--	[35]
11.	Glyceryl monostearate	fatty acid ester	24699	0.33	--	[29]
12.	Phytol	terpenoid	5280435	0.27	[36]	--
13.	4-Vinylphenol	phenol	62453	0.25	[26]	[30]
14.	Squalene	terpenoid	638072	0.21	--	--
15.	Benzoic acid	carboxylic acid	243	0.17	[27]	[28]
16.	cis-cinnamic acid	carboxylic acid	5372954	0.14	[19]	--
17.	Phenol	phenol	996	0.07	[26]	--
18.	Eicosane	alkane	8222	0.07	--	[37]

**Table 4:** Particle size analysis and polydispersity index of both AE and ME extract-based silver nanoparticles.

Extract	Light Intensity (Lumens)	Percent Nanoparticles Yield	Z- Avg. (d. nm)	PDI	Size (d. nm)
Aqueous	dark	17.26% ± 0.06	55.53 ± 8.547	0.268	28.21
	250	12.04% ± 0.57	61.58 ± 0.386	0.378	4.765
	825	8.63% ± 0.57	66.93 ± 11.19	0.152	39.81
Methanol	Dark	1.23% ± 0.06	48.43 ± 6.282	0.315	20.90
	250	4.52% ± 0.141	68.10 ± 11.90	0.153	44.63
	825	7.41% ± 0.14	75.19 ± 14.58	0.239	47.49

**Table 5:** Percentage elemental analysis of silver nanoparticles by energy-dispersive X-ray (EDX) spectroscopy.

Extract	Light Intensity	% Silver	% Carbon	% Nitrogen	% Oxygen
Aqueous	Dark	18.16 ± 1.01	11.50 ± 0.36	5.30 ± 0.2	31.96 ± 2.15
	825	13.80 ± 0.78	16.60 ± 0.65	2.46 ± 0.46	5.70 ± 0.26
Methanol	Dark	24.16± 5.23	18.40 ± 0.20	3.53 ± 0.30	25.06 ± 1.50
	825	14.16 ± 2.34	20.76 ± 2.12	2.10 ± 0.36	24.46 ± 6.00

**Table 6:** Functional group analysis of the physical mixture of the extract and AgNO_3_ and silver nanoparticles by Fourier transform infrared analysis.

Wave Numbers (cm^−1^)						Vibration Type
AE Based AgNPs			ME Based AgNPs			
Physical Mixture	Dark	825 Lumens	Physical Mixture	Dark	825 Lumens	
3399.8	--	--	--	3435	--	O-H stretching and N = H stretching (presence of primary amine group)
--	2963.3	2963.2	--	2963.3	--	
1613.8			--	--	--	C––C Aromatic stretch
1384.1	--	--	1383.4	--	--	NH_2_ rocking
1261.2	1261.4	1261.5	--	1261.5	1261.4	C–O stretching vibrations of alcohols, ethers, esters, carboxylic acids
1090.8	1095	1095	--	1095.8	1094.8	C-O, C-C band of glycosides
799.8	800.9	800.9	801	800.9	800.6	C-H band of alkanes

## Data Availability

Data is contained within the article.
